# Supplementary material for: Incorporating mutational heterogeneity to identify genes that are enriched for synonymous mutations in cancer
Source: BMC Bioinformatics. 2023 Dec 7;24:462. doi: 10.1186/s12859-023-05521-8 (PMC10704839; doi:10.1186/s12859-023-05521-8)
Supplement: Supplementary file 1 — Additional file 1. Figure S1. MutSigCVsyn non-synonymous cancer driver landscape: Heatmap displaying 133 significant nonsynonymous candidate genes (Benjamini-Hochberg FDR < 1× 10^-2^ ) identified by MutSigCVsyn. Candidate genes are divided into two columns and are ranked from most frequent across all histology cohorts (left top) to the least frequent ones (right bottom). Candidate genes are colored by negative logarithmic transformed FDR value from high (dark blue) to low (light yellow). Figure S2. Potential functional role of MutSigCVsyn candidate genes: (A) Boxplot of Breast-AdenoCA patient PURA mRNA expression level of normal samples and tumor samples. The P-value is calculated by the Mann-Whitney U test. (B) Boxplot of SIGLEC15 expression data from DepMap Pancreas exocrine cell lines and all other tested cell lines. The P-values are calculated by the Mann-Whitney U test. (C) CADD analysis of synonymous mutations in synonymous candidate genes against the ones in all other genes. The P-values are calculated by the Mann-Whitney U test. Figure S3. MutSigCVsyn identifies PCAWG-exclusive drivers in non-synonymous analysis: PCAWG-exclusive drivers are the cancer driver genes that were first identified by the PCAWG working group. There are in total 15 exclusive non-synonymous protein-coding drivers in PCAWG and they are shown in the table. The ‘gene’ column shows the gene name. ‘cds’ in the ’Element_type’ column shows that the coding region of the gene is identified as a cancer driver. ‘discovery_unique’ in the ‘category’ column shows that the gene is first identified by PCAWG. 6 of them (highlighted yellow) were identified by MutSigCVsyn in non-synonymous mutation analysis. [file 12859_2023_5521_MOESM1_ESM.pdf]

FigureS1

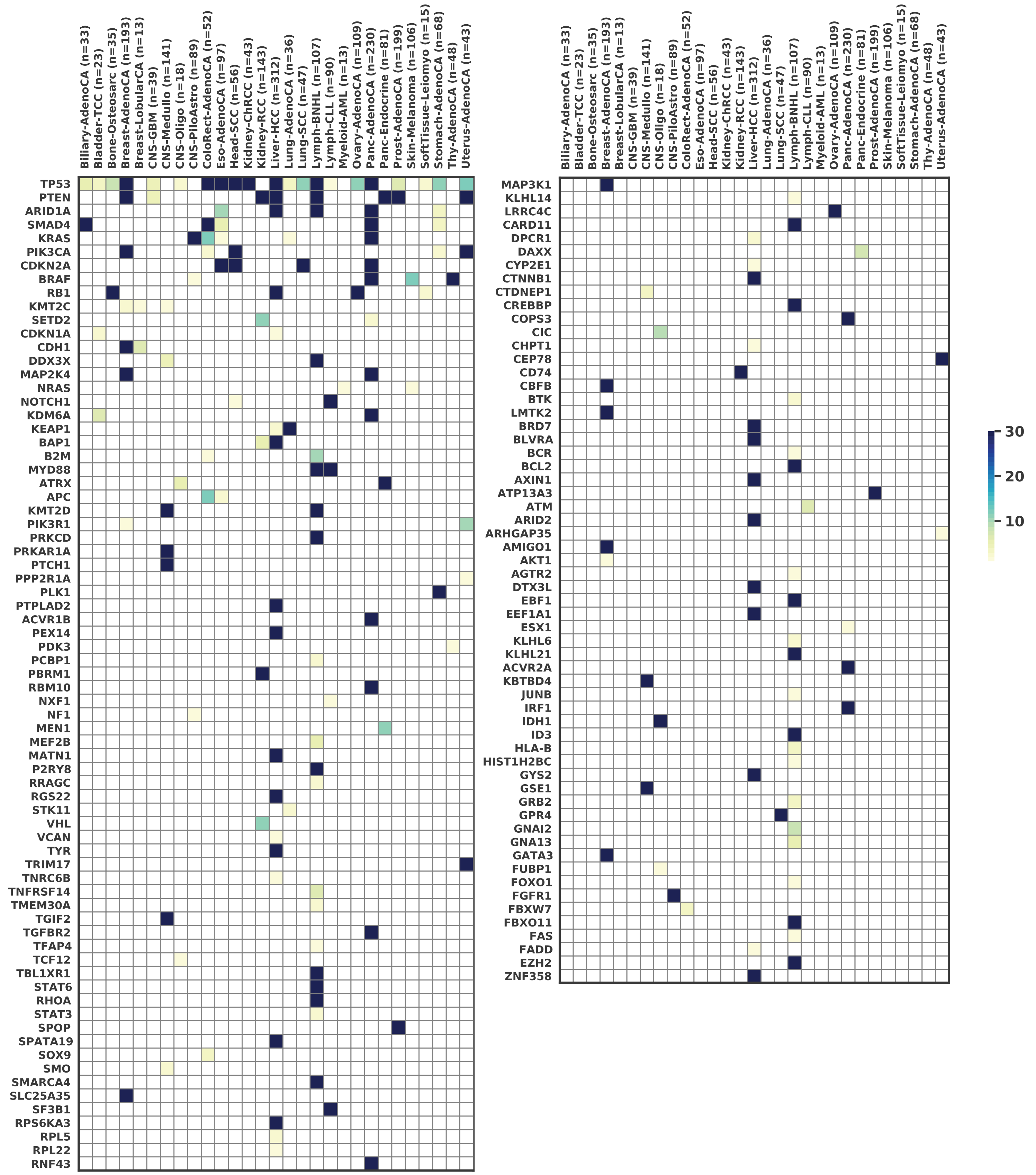
Biliary-AdenoCA (n=33)
Bladder-TCC (n=23)
Bone-Osteosarc (n=35)
Breast-AdenoCA (n=193)
Breast-LobularCA (n=13)
CNS-GBM (n=39)
CNS-Medullo (n=141)
CNS-Oligo (n=18)
CNS-PiloAstro (n=89)
ColoRect-AdenoCA (n=52)
Eso-AdenoCA (n=97)
Head-SCC (n=56)
Kidney-ChRCC (n=43)
Kidney-RCC (n=143)
Liver-HCC (n=312)
Lung-AdenoCA (n=36)
Lung-SCC (n=47)
Lymph-BNHL (n=107)
Lymph-CLL (n=90)
Myeloid-AML (n=13)
Ovary-AdenoCA (n=109)
Panc-AdenoCA (n=230)
Panc-Endocrine (n=81)
Prost-AdenoCA (n=199)
Skin-Melanoma (n=106)
SoftTissue-Leiomyo (n=15)
Stomach-AdenoCA (n=68)
Thy-AdenoCA (n=48)
Uterus-AdenoCA (n=43)
TP53
PTEN
ARID1A
SMAD4
KRAS
PIK3CA
CDKN2A
BRAF
RB1
KMT2C
SETD2
CDKN1A
CDH1
DDX3X
MAP2K4
NRAS
NOTCH1
KDM6A
KEAP1
BAP1
B2M
MYD88
ATRX
APC
KMT2D
PIK3R1
PRKCD
PRKAR1A
PTCH1
PPP2R1A
PLK1
PTPLAD2
ACVR1B
PEX14
PDK3
PCBP1
PBRM1
RBM10
NXF1
NF1
MEN1
MEF2B
MATN1
P2RY8
RRAGC
RGS22
STK11
VHL
VCAN
TYR
TRIM17
TNRC6B
TNFRSF14
TMEM30A
TGIF2
TGFBR2
TFAP4
TCF12
TBL1XR1
STAT6
RHOA
STAT3
SPOP
SPATA19
SOX9
SMO
SMARCA4
SLC25A35
SF3B1
RPS6KA3
RPL5
RPL22
RNF43
MAP3K1
KLHL14
LRRC4C
CARD11
DPCR1
DAXX
CYP2E1
CTNNB1
CTDNEP1
CREBBP
COPS3
CIC
CHPT1
CEP78
CD74
CBFB
BTK
LMTK2
BRD7
BLVRA
BCR
BCL2
AXIN1
ATP13A3
ATM
ARID2
ARHGAP35
AMIGO1
AKT1
AGTR2
DTX3L
EBF1
EEF1A1
ESX1
KLHL6
KLHL21
ACVR2A
KBTBD4
JUNB
IRF1
IDH1
ID3
HLA-B
HIST1H2BC
GYS2
GSE1
GRB2
GPR4
GNAI2
GNA13
GATA3
FUBP1
FOXO1
FGFR1
FBXW7
FBXO11
FAS
FADD
EZH2
ZNF358
30
20
10

FigureS2

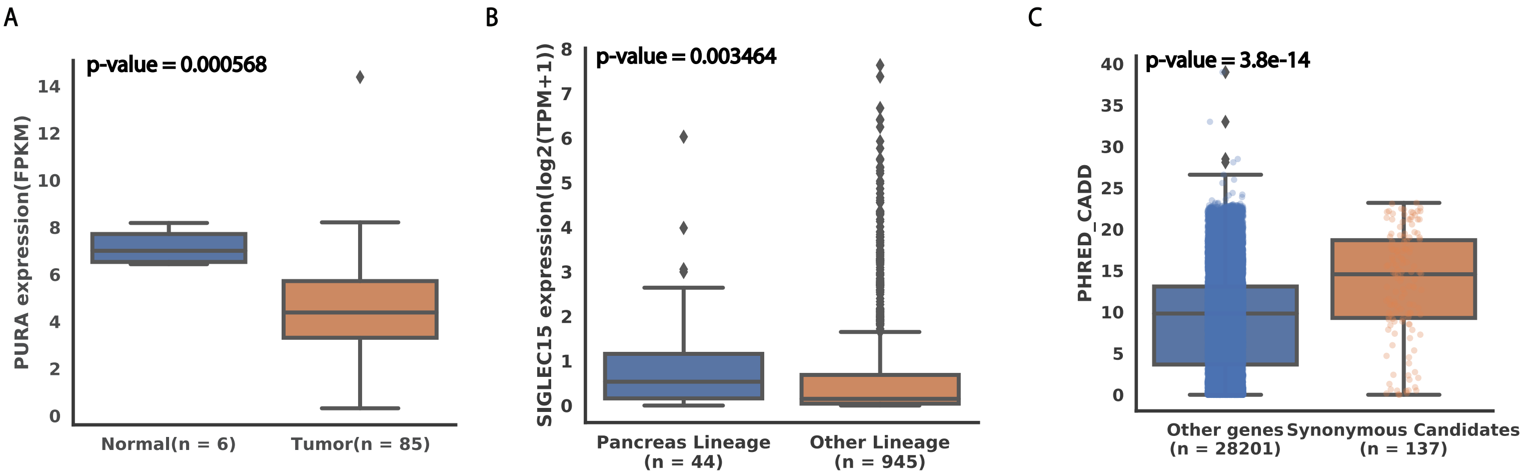
A
p-value = 0.000568
PURA expression(FPKM)
0
2
4
6
8
10
12
14
Normal(n = 6)
Tumor(n = 85)
B
p-value = 0.003464
SIGLEC15 expression(log2(TPM+1))
0
1
2
3
4
5
6
7
8
Pancreas Lineage
(n = 44)
Other Lineage
(n = 945)
C
p-value = 3.8e-14
PHRED_CADD
0
5
10
15
20
25
30
35
40
Other genes
(n = 28201)
Synonymous Candidates
(n = 137)

Figure S3

Gene	Element_type	Category
TMEM30A	cds	discovery_unique
PLK1	cds	discovery_unique
PA2G4	cds	discovery_unique
SRSF7	cds	discovery_unique
CAMK1	cds	discovery_unique
TMSB4X	cds	discovery_unique
KLHL6	cds	discovery_unique
RRAGC	cds	discovery_unique
GRB2	cds	discovery_unique
DYRK1A	cds	discovery_unique
CTC-512J12.6	cds	discovery_unique
DYNC1I1	cds	discovery_unique
PRKCD	cds	discovery_unique
RIPK4	cds	discovery_unique
RELA	cds	discovery_unique
