# Supplementary material for: Incorporating mutational heterogeneity to identify genes that are enriched for synonymous mutations in cancer
Source: BMC Bioinformatics. 2023 Dec 7;24:462. doi: 10.1186/s12859-023-05521-8 (PMC10704839; doi:10.1186/s12859-023-05521-8)
Supplement: Supplementary file 2 — Additional file 2. Detailed methods and results of mutual exclusivity and co-occurrence analysis, CADD analysis, and gProfiler analysis. [file 12859_2023_5521_MOESM2_ESM.docx]

Mutual Exclusivity and Co-occurrence Analysis

Driver genetic alterations are less likely to occur with another that has a similar functional effect in the same molecular pathway[(1)](https://paperpile.com/c/YzcEks/73SI). To examine the driver potential of our synonymous mutations, we performed a one-sided Fisher's exact test on synonymous driver genes against known cancer drivers in PCAWG across all cohorts (Additional File 3: Table S1).

However, after multiple hypothesis corrections, we did not identify any mutually exclusive pairs in our analysis. This lack of findings can primarily be attributed to the small sample size. On average, only 3.6% of the patients harbor synonymous mutations, and 11% of the patients with non-synonymous alterations. We did a simple power analysis and discovered that, given the average percentage of patients harboring synonymous or nonsynonymous alternations, to reach 80% detecting power for mutual exclusivity pairs, at least 1200 patients are required. (odds ratio = 0.5, contingency table simulated from multinomial distribution). Therefore, due to the smaller mutational frequency of synonymous mutations, further investigation and future studies with larger sample sizes might help to increase statistical power and thus clarify the role of synonymous candidates.

Besides mutual exclusivity, the co-occurrence of drivers, which implies that the alteration of both driver genes occurs together more often than expected, is another key phenomenon. A typical example is that oncogenic KRAS mutations in non-small cell lung cancer patients often co-occur with mutations in STK11 and KEAP1[(2)](https://paperpile.com/c/YzcEks/x9AAY), the former relieves the repression of squamous cell differentiation genes by down-regulating Polycomb Repressive Complex2, the latter regulates cellular antioxidant, metabolic and anti-inflammatory pathways by regulating transcription factor NRF2. Alternations in all these genes contribute to a more aggressive NSCLC type. However, co-occurrence of drivers is much less frequently observed in cancer.

Interestingly, our analysis did point toward a likelihood of co-occurrence for BCL-2 and CREBBP in the Lymphoma cohort (q-value = ​​8.14E-5 )(Additional File 3: Table S3). This co-occurrence has been identified and validated in transgenic mice, where the CREBBP deletion and BCL2 overexpressed mouse strain have much worse lymphoma-specific survival than each of the alterations alone[(3)](https://paperpile.com/c/YzcEks/mNcPc). In the study, deletion of CREBBP was found to be associated with increased myc expression, which is another major driver in lymphoma. The observation that PCAWG patients with synonymous mutations in BCL2 are more likely to harbor CREBBP mutation as well, supports our hypothesis in the paper that synonymous mutations may enhance BCL2 expression in the Lymphoma cohort.

Combined Annotation Dependent Depletion (CADD) analysis

To elucidate the functional impact of our synonymous candidates, we employed CADD (Combined Annotated Dependent Depletion)[(4)](https://paperpile.com/c/YzcEks/u7G2X). CADD utilizes diverse genomic annotations, including conservation scores, splicing prediction, sequence context, and population-level sequence information, to estimate the likelihood of a variant being deleterious. CADD allowed us to evaluate the potential functional consequences of synonymous mutations, considering their effects on conservation, splicing, and population frequency.

We compared the CADD prediction for the synonymous mutation in our predicted driver genes to the ones in other genes. With CADD, we obtained strongly positive results, indicating that synonymous mutations in our candidate genes are significantly more deleterious compared to other genes (p = 3X10^-14, n = 137) (Figure S2C). This result supports the significance of the functional relevance of these synonymous drivers.

gProfiler Analysis

We performed gProfiler analysis[(5)](https://paperpile.com/c/YzcEks/HLGm) to explore the functional enrichment of our candidate genes (Additional File 3: Table S5). At highly stringent p-value cutoffs (10^-16), no effects were observed, however, at p-values of around 10^-2 to 10^-3 we did see effects. The pathway related terms are: TROP2 regulatory pathway (p-value = 4.5X10^-3, including BCL2, ACVRL1 and ADAM17), and Response to hypoxia pathway(p-value = 4.2^10-2, including BCL2, RTN4, ITPR2, ACVRL1, ADAM7). Both pathways are deeply associated with cancer cell-specific proliferation[(6,7)](https://paperpile.com/c/YzcEks/ljU7+ld6C). Therefore, these genes may collectively contribute to enhancing cancer cell proliferation.

References

1. [El Tekle G, Bernasocchi T, Unni AM, Bertoni F, Rossi D, Rubin MA, et al. Co-occurrence and mutual exclusivity: what cross-cancer mutation patterns can tell us. Trends Cancer Res. 2021 Sep;7(9):823–36.](http://paperpile.com/b/YzcEks/73SI)

2. [Skoulidis F, Heymach JV. Co-occurring genomic alterations in non-small-cell lung cancer biology and therapy. Nat Rev Cancer. 2019 Sep;19(9):495–509.](http://paperpile.com/b/YzcEks/x9AAY)

3. [García-Ramírez I, Tadros S, González-Herrero I, Martín-Lorenzo A, Rodríguez-Hernández G, Moore D, et al. loss cooperates with overexpression to promote lymphoma in mice. Blood. 2017 May 11;129(19):2645–56.](http://paperpile.com/b/YzcEks/mNcPc)

4. [Rentzsch P, Witten D, Cooper GM, Shendure J, Kircher M. CADD: predicting the deleteriousness of variants throughout the human genome. Nucleic Acids Res. 2019 Jan 8;47(D1):D886–94.](http://paperpile.com/b/YzcEks/u7G2X)

5. [Raudvere U, Kolberg L, Kuzmin I, Arak T, Adler P, Peterson H, et al. g:Profiler: a web server for functional enrichment analysis and conversions of gene lists (2019 update). Nucleic Acids Res. 2019 Jul 2;47(W1):W191–8.](http://paperpile.com/b/YzcEks/HLGm)

6. [Shvartsur A, Bonavida B. Trop2 and its overexpression in cancers: regulation and clinical/therapeutic implications. Genes Cancer. 2015 Mar;6(3-4):84–105.](http://paperpile.com/b/YzcEks/ljU7)

7. [Muz B, de la Puente P, Azab F, Azab AK. The role of hypoxia in cancer progression, angiogenesis, metastasis, and resistance to therapy. Hypoxia (Auckl). 2015 Dec 11;3:83–92.](http://paperpile.com/b/YzcEks/ld6C)
